# Plant-Based Nanovesicular Gel Formulations Applied to Skin for Ameliorating the Anti-Inflammatory Efficiency

**DOI:** 10.3390/gels10080525

**Published:** 2024-08-10

**Authors:** Hanan Abdelmawgoud Atia, Mona M. Shahien, Somaia Ibrahim, Enas Haridy Ahmed, Hemat A. Elariny, Marwa H. Abdallah

**Affiliations:** 1 Department of Pharmacology and Toxicology, College of Pharmacy, University of Ha’il, Ha’il 81442, Saudi Arabia; h.soliman@uoh.edu.sa (H.A.A.); hem.mohammed@uoh.edu.sa (H.A.E.); 2 Department of Biochemistry, Faculty of Pharmacy, Al-Azhar University, Cairo 11651, Egypt; 3 Department of Pediatrics, College of Medicine, University of Ha’il, Ha’il 81442, Saudi Arabia; m.shahin@uoh.edu.sa (M.M.S.); so.bashir@uoh.edu.sa (S.I.); 4 Department of Anatomy, College of Medicine, University of Ha’il, Ha’il 81442, Saudi Arabia; e.haridy@uoh.edu.sa; 5 Department of Anatomy and Embryology, Faculty of medicine, Ain Shams University, Cairo 11566, Egypt; 6 Department of Pharmacology and Toxicology, Faculty of Pharmacy, Al-Azhar University, Cairo 11651, Egypt; 7 Department of Pharmaceutics, College of Pharmacy, University of Ha’il, Ha’il 81442, Saudi Arabia; 8 Department of Pharmaceutics, Faculty of Pharmacy, Zagazig University, Zagazig 44519, Egypt

**Keywords:** natural products, inflammation, nanovesicular gel, skin, drug delivery

## Abstract

Inflammation is a vascular response that occurs when the immune system responds to a range of stimuli including viruses, allergens, damaged cells, and toxic substances. Inflammation is accompanied by redness, heat, swelling, discomfort, and loss of function. Natural products have been shown to have considerable therapeutic benefits, and they are increasingly being regarded as feasible alternatives for clinical preventative, diagnostic, and treatment techniques. Natural products, in contrast to developed medications, not only contain a wide variety of structures, they also display a wide range of biological activities against a variety of disease states and molecular targets. This makes natural products appealing for development in the field of medicine. In spite of the progress that has been made in the application of natural products for clinical reasons, there are still factors that prevent them from reaching their full potential, including poor solubility and stability, as well limited efficacy and bioavailability. In order to address these problems, transdermal nanovesicular gel systems have emerged as a viable way to overcome the hurdles that are encountered in the therapeutic use of natural products. These systems have a number of significant advantages, including the ability to provide sustained and controlled release, a large specific surface area, improved solubility, stability, increased targeting capabilities and therapeutic effectiveness. Further data confirming the efficacy and safety of nanovesicles–gel systems in delivering natural products in preclinical models has been supplied by extensive investigations conducted both in vitro and in vivo. This study provides a summary of previous research as well as the development of novel nanovesicular gel formulations and their application through the skin with a particular emphasis on natural products used for treatment of inflammation.

## 1. Introduction

Inflammation is a protective reaction of the body to harmful substances like allergens, radiation, toxic compounds or tissue damage. It acts by removing injurious stimuli and initiating the healing process [1,2]. Typically, in acute inflammatory reactions, cellular and molecular processes effectively reduce the risk of injury or infection. This process of modulation helps restore tissue balance and resolve acute inflammation. Nevertheless, if acute inflammation is not properly controlled, it can progress into a chronic state, which can then contribute to the development of various chronic inflammatory diseases [3]. It can lead to a wide range of disorders such as allergies, cardiovascular problems, metabolic syndrome, cancer, and autoimmune diseases [4]. Inflammation at the tissue level is identified by swelling, redness, pain, heat, and impaired tissue function. These symptoms occur due to the immune system, blood vessels, and inflammatory cells responding to infection or injury [5]. Essential microcirculatory events that take place during the inflammatory process involve alterations in vascular permeability, the recruitment and buildup of leukocytes, and the release of inflammatory mediators [6].

**Figure 1:**
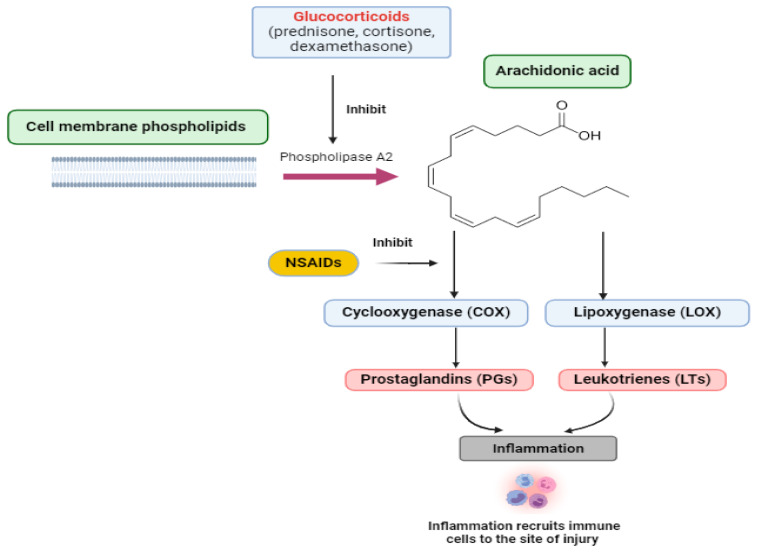
Arachidonic acid pathway and its inhibition by several drugs.

When a cell is harmed, phospholipids and other substances are released from the cell. Phospholipids trigger the arachidonic acid chain reaction, in which cyclooxygenase and 5-lipooxygenase produce prostaglandins and leukotrienes, respectively, as depicted in Figure 1. Arachidonic acid is the most crucial precursor of inflammatory pathways. Figure 1 illustrates the suppression of the arachidonic acid pathway by multiple anti-inflammatory medications. Corticosteroids (glucocorticoids) and nonsteroidal anti-inflammatory drugs (NSAIDs) are frequently employed for the treatment of inflammation as well as for the alleviation of pain and fever [7]. Corticosteroids inhibit the phospholipase-A_2_ enzyme, preventing the synthesis of arachidonic acids and their subsequent products, including cyclooxygenase (COX) and lipoxygenase (LOX). On the other hand, nonsteroidal anti-inflammatory drugs (NSAIDs) specifically inhibit the COX pathway. There are two distinct isoforms of COX, known as COX-1 and COX-2, which are involved in different functions and have different gene codes [8,9]. COX-2 predominantly contributes to detrimental inflammatory reactions, while COX-1 is associated with homeostatic functions. As a result, researchers developed selective COX-2 inhibitors [9,10]. The development of novel nonsteroidal anti-inflammatory drugs (NSAIDs) that are specific for (COX-2) seems to be the most promising strategy, while COX-1 inhibition leads to unfavorable side effects. These type of specific NSAIDS (COX-2 inhibitors) inhibit the COX-2 enzyme pathway, which leads to a redirection of arachidonic acid metabolism to the other main lipoxygenase (LOX) pathway. This redirection increases the production of leukotrienes (LT) and triggers an inflammatory response [11].

Steroids suppress the production of arachidonic acid, thus preventing the synthesis of leukotrienes and prostaglandins. Nonsteroidal anti-inflammatory drugs inhibit the synthesis of 5-lipoxygenase and cyclooxygenase and inhibit the conversion of cyclo-oxygenase to prostaglandins and thromboxane A_2_. Four main bioactive prostaglandins are produced in vivo: PGE_2_, PGI_2_ (prostacyclin), PGD_2_, and PGF_2_α. Most cell types produce one or two major products, which operate as autocrine and paracrine lipid mediators to maintain local homeostasis. The amount and profile of prostaglandin synthesis alter drastically during inflammation. Prostaglandin synthesis is typically quite low in non-inflammatory tissues, but it quickly rises in acute inflammation before leukocyte recruitment and immune cell infiltration [12].

There are several medications available to control and reduce inflammation conditions. Steroids, nonsteroidal anti-inflammatory drugs, and immune suppressants are examples of these medications. However, they can have negative side effects. Due to the notable adverse effects of steroidal and NSAID medications, there is a growing prevalence toward natural compounds, such as dietary supplements and herbal remedies, which have been historically employed for centuries to alleviate pain and inflammation [13]. Several of these natural compounds also function by suppressing the inflammatory pathways in a comparable way to NSAIDs. Aside from the COX pathway, numerous natural compounds function to hinder the nuclear factor-kB (NF-kB) inflammatory pathways. The NF-kB molecule is a transcription factor that regulates the transcription of DNA to maintain the ongoing inflammatory immune response.

Plant components have historically been utilized in traditional treatment and as precursors for the creation of medicines. The efficacy of the majority of bioactive components found in phytomedicines is hindered by their inadequate systemic absorption whether taken orally or applied topically. Herbal extracts face several challenges, such as physicochemical factors that can lead to low and inconsistent solubility, chemical instability in acidic pH and metabolism, and limited permeation through the cell membrane and restricted biological distribution or rapid metabolic clearance [14,15,16]. The reduced efficacy of bioactive components in phytomedicines, due to inadequate absorption into the bloodstream, can be ascribed to many variables, such as low bioavailability, poor therapeutic effectiveness, first-pass metabolism, low lipid solubility, efflux transporters, and chemical instability [17,18]. In addition, chemicals with large molecular weight and low lipophilicity face difficulties in permeating and being absorbed by biological membranes and barriers [19,20]. Therefore, there has been a focused endeavor to create nanovesicular drug delivery systems that can transport both hydrophilic and hydrophobic substances and achieve concentrations that are meaningful in a clinical setting [15]. Consequently, current research is primarily concentrated on the development of innovative carrier systems, such as surfactant and polymer/lipid-based systems, to enhance the efficient delivery of plant-based constituents.

Treatment using a transdermal drug delivery system is one of the least invasive and patient-friendly methods. It concentrates medicine molecules in a skin area to increase absorption and reduce side effects. Transdermal drug delivery is a promising oral and hypodermic injection alternative [21]. Thus, developing novel TDDs, improving drug penetration over the skin barrier, and reaching therapeutic drug concentrations in target cutaneous tissues is greatly requested.

Our goal is to provide a summary of previous research as well as the development of novel nanovesicular gel formulations based on using the drug lowest effective dose that provides the highest effectiveness while causing the fewest adverse effects. Therefore, it is necessary to incorporate natural anti-inflammatory elements into prescription therapy in order to enhance the pharmacological response and minimize the occurrence of undesirable side effects [22,23].

## 2. Nanovesicular Drug Delivery Systems

Nanovesicular drug delivery systems are novel formulations that employ vesicles to encapsulate and convey medications to precise destinations within the body. Vesicular drug delivery systems refer to a type of drug delivery system that involves the use of vesicles to transport drugs to specific target sites in the body. Nanovesicles have garnered attention for treating skin problems in recent decades. Nanovesicles penetrate skin layers to treat diseases. They deliver drugs site-specifically. Conventional therapies treatments are long-lasting, have adverse effects, and weaken the skin barrier. Nanovesicles improve medication deposition and decrease negative effects [24].

Nanovesicles are colloidal particles formed by surrounding an aqueous phase with amphoteric molecules like phospholipids or surfactants, resulting in one or more concentric lipid bilayers [25]. Vesicles have the ability to contain lipophilic medications inside the lipid bilayer or hydrophilic drugs within the aqueous core [26]. They possess the capability to transport medications to the specific site of action, hence reducing the possibility of drug toxicity [27]. Moreover, vesicular delivery techniques have been proven to be more effective than traditional systems [28]. Research has shown that vesicles have the ability to improve the absorption of anti-inflammatory medications into the skin. This is because the lipidic components of vesicles may penetrate the outermost layer of the skin, stratum corneum, and alter its lipid structure [29].

**Figure 2:**
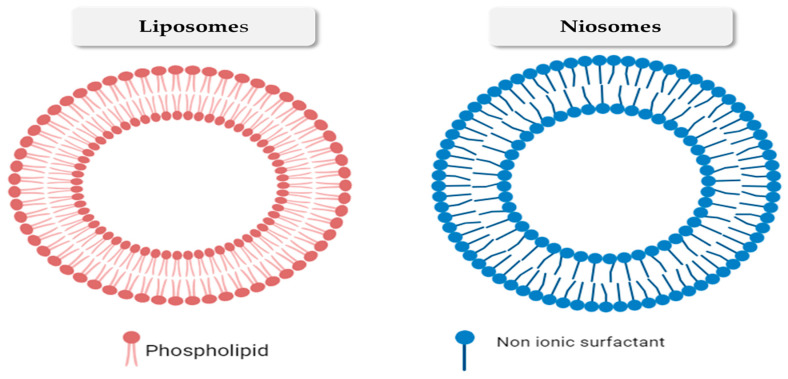
Schematic diagram represents the structure of the conventional nanovesicles (liposomes and niosomes).

Liposomes and niosomes (Figure 2) are recognized as the first identified vesicles [30]. Liposomes are small spherical vesicles composed of lipid bilayers (phospholipids and cholesterol) with a diameter ranging from 0.05 to 5.0 μm. The term “liposome” is derived from the Greek words “lipos” meaning fat and “soma” meaning body [31]. Due to their hydrophilic and lipophilic characteristics, they are very suitable for drug administration in various structures, making them a very promising option [32,33]. This drug delivery technique aims to precisely target the pharmacologically active compounds to the intended site of action [34]. Liposomes possess a special characteristic that allows them to trap hydrophilic and lipophilic compounds within their compartments and provide a controlled-release effect. They are also biocompatible, biodegradable, and stable [33,35].

**Figure 3:**
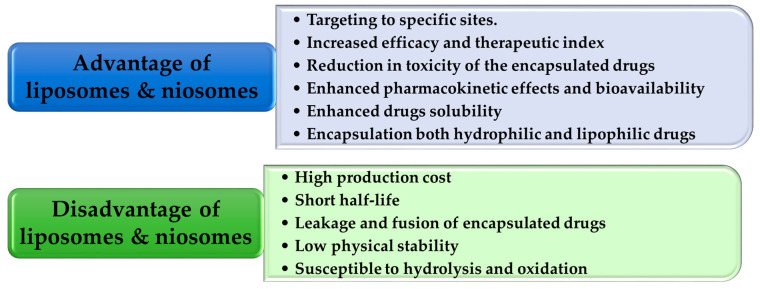
The advantages and disadvantages of traditional nanovesicular systems [25,43].

Niosomes are small vesicles with a size in the nanometer range, which are created by mixing a non-ionic surfactant with a lipid-like substance called cholesterol [36]. The hydrophobic regions in the bilayer structure are repelled away from the aqueous side, while the hydrophilic heads remain in connection with the aqueous phase. To ensure the safety of the niosomes, the surfactants utilized in their manufacture should be biodegradable, biocompatible and non-immunogenic [37]. Niosomes function similarly to liposomes both in vivo and in vitro, prolonging the duration of action of the entrapped drugs, modifying their distribution throughout organs, and enhancing their bioavailability. The niosomal formulations exhibit higher permeability compared to liposomes with equivalent cholesterol content. Prior studies have demonstrated that the concentration of cholesterol has a significant role in influencing vesicle leakage [38]. Consequently, the efficacy of liposomal drug entrapment is reduced compared to niosomes [39]. Liposomes are costly, because their constituents are prone to instability over extended periods and require specialized handling and storage [40,41]. In addition, they have some drawbacks stemming from their inadequate physical stability, which can result in aggregation, leakage, and the merging of bioactive compounds, as demonstrated in Figure 3. These limitations restrict their practicability and effectiveness [42]. Recently, a number of innovative vesicular systems have been employed in order to address these constraints.

**Figure 4:**
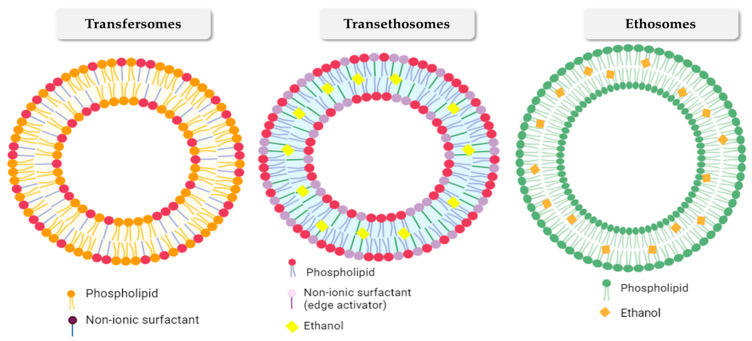
Schematic representation of the structure of various innovative nanovesicular carriers.

Figure 4 illustrates the structure of various innovative nanovesicular systems. The composition of the vesicle is a crucial component that influences its ability to penetrate the layers of the skin. Several researchers have documented different carrier systems that include various components. These systems are crucial for overcoming skin barriers and improving the penetration through the stratum corneum. This review will examine the facts of new nanovesicular carriers for delivering anti-inflammatory plant-based compounds via the skin, as shown in Table 1.

### 2.1. Transfersomes

Transfersomes are biodegradable and biocompatible vesicles that were first described in the 1990s by Cevc et al. The structure of transfersomes includes an edge activator and phospholipids [66], as seen in Figure 4. The edge activator offers the vesicles ultra-deformable properties known as self-optimizing deformability, which enables the transfersomes to change their flexibility and allow transport through the skin’s pores [67,68]. Furthermore, the enhanced transportation of drugs by transfersomes is facilitated by the osmotic gradient between the outer and inner layers of the stratum corneum. This gradient enables transfersomes to penetrate the intact skin through the natural transcutaneous hydration gradient. Transfersomes readily accumulate within the permeable synovial tissue, leading to peripheral targeting. Additionally, they serve as storage sites, leading to the creation of a controlled drug delivery system [69,70]. Nevertheless, transfersomes exhibit considerable manufacturing expense and limited chemical stability resulting from their ability to oxidative degradation. The difficulty of achieving the purity of natural phospholipids is another obstacle to using transfersomes as a drug delivery system; consequently, synthetic phospholipids have the potential to serve as substitutions [71]. Various formulations based on transfersomes are presently undergoing evaluation in various phases of clinical trials. When it comes to treating knee osteoarthritis, for instance, a phase III clinical trial was used to examine the safety and effectiveness of ketoprofen in transfersomes (Diractin^®^). After six weeks of treatment, the drug in its transfersomal carrier was found to be more effective than a placebo in reducing pain from knee osteoarthritis with fewer side effects [72]. Although the market formula had great clinical effectiveness and outcomes, it was withdrawn from the market due to its excessive cost.

Wu et al. studied the utilization of novel carriers called transfersomes to encapsulate resveratrol (RSV) to assess its anti-inflammatory and its antioxidant responses [44]. Phosphatidyl choline and various non-ionic edge activators (Tween 80, Plantacare^®^ 1200 UP, Tween 20) were used to develop transfersomes using high-pressure homogenization techniques. Edge activators play a significant role in transfersomes manufacturing because they have the ability to increase the lipid membrane’s flexibility. Moreover, the drug can pass through the stratum corneum and into the skin because of its ultra-deformability. The results demonstrated that resveratrol’s antioxidant activity is unaffected by the encapsulation process, since transfersomes exhibited antioxidant activity that was similar to resveratrol. Consequently, transfersomes were able to enhance RSV’s safety, solubility, and stability. Patel et al. generated curcumin-loaded transfersomes for evaluating the transdermal delivery of curcumin [45]. Transfersomes were developed using a modified hand shaking, lipid film hydration technique. The optimum formulation was chosen by examining different process variables such as the lecithin/surfactant ratio and type of surfactant (tween 80 and span 80). The optimum transfersomal formulation showed the maximum entrapment efficiency and provided a higher permeation of drug from the developed transfersomal gel. Consequently, the transfersomal formulation is preferable for transdermal curcumin administration. In addition, transfersomes-encapsulated curcumin represents a promising strategy for targeted anti-inflammatory therapy. By overcoming the limitations of poor solubility and bioavailability associated with curcumin, transfersomes offer a potential solution for improving the treatment outcomes of inflammatory disorders.

Umam et al. formulated sesamol-loaded transfersomal gel for delayed wound healing. Sesamol-loaded transfersomes were prepared using thin-film hydration [46]. Optimized formulations with Tween-80 and Span-80 edge activators were created. Vesicles were evaluated for their size, shape, loading efficiency, deformability, and in vitro skin penetration. The optimized formulation was added to 1% carbopol 940 gel to produce sesamol-loaded transfersomal gel. Better skin penetration and deposition were observed in sesamol-loaded transfersomal gel. Research on wound healing has shown that transfersomes loaded with sesamol result in the greatest amount of wound contraction. The transfersomal gel treatment led to a significant improvement in skin histological architecture after 21 days. The results of the study indicated that transfersomal gel might be a promising alternative for wound-healing treatments

Pandit et al. generated quercetin as deformable transfersomes topically to treat osteoporosis in order to improve its limited oral solubility and bioavailability [47]. The transfersomes formulation technique was investigated using fractional factorial design. Transfersomes had excellent entrapment, particle size, zeta potential, and polydispersity index. After loading transfersomes into chitosan film, they demonstrated excellent penetration into rat skin. The study concluded that quercetin-loaded transfersomes chitosan film reduced osteoclastogenesis and osteoblast apoptosis, leading to increased osteoblast numbers and bone mineralization. Chitosan film with quercetin-loaded transfersomes is an effective alternative to oral quercetin administration for treating osteoporosis, resulting in enhanced patient compliance.

Rizvi et al. developed a Berberis aristata nanovesicular gel and evaluated its anti-inflammatory and antipsoriatic properties [48]. Transfersomes were generated utilizing soya phosphatidylcholine and edge activators (Tween 80, Span 80, and sodium deoxycholate) using a rotary evaporator by the modified lipid film hydration process and assessed it for different parameters. Transfersomes exhibited a reasonable vesicle size and entrapment efficiency. Transfersomes were added to Carbopol 934P to produce gels and tested them for physicochemical characteristics. Their effectiveness against inflammation, IMQ-induced psoriasis, and skin irritation was compared to commercial product (Angle Gloss (BHARGAVA, Phytolab Pvt. Ltd., Noida, India). By the carrageenan-induced paw edema technique, transferosomal gel (55.76%) inhibited edema better than traditional gel of the extract (33.5%). Histopathological analysis demonstrated that extract-loaded transfersomal gel reduced epidermal thickness in psoriasis-induced animals compared to traditional gel formulation. Generally, *Berberis aristata* extract-loaded transfersomal gel is considered a promising system for the effective anti-inflammatory and antipsoriatic activities. 

### 2.2. Ethosomes

Ethosomes are nanovesicular carriers with high alcohol content created from hydroalcoholic phospholipid. Ethosomes are soft, flexible vesicles that carry medications to deep skin layers and/or the systemic circulation. Due to their extreme flexibility, ethosomes have a remarkable capacity to penetrate human skin. Ethosomes range in size from nanometers to microns [73]. Ethosomes are modified liposomes with high ethanol concentration. In the ethosomal system, phospholipids (phosphatidylglycerol, phosphatidylcholine, phosphatidylinositol, phosphatidic acid, phosphatidylserine, and phosphatidylethanolamine), high alcohol concentrations (ethanol and isopropyl alcohol), and water are present [74,75]. Ethanol’s high concentration in ethosomes disrupts skin lipid bilayer construction, enhancing vesicle membrane penetration into the stratum corneum [76]. Ethosomes are commonly used topically due to their excellent drug transport over the skin barrier [77]. Since ethosomes include naturally occurring phospholipids, their purity could cause a concern [25] in addition to skin irritation caused by the high content of alcohol. Moreover, ethosomal vesicles may exposed to aggregation and precipitation [78]. Drugs can be trapped in ethosomes with hydrophilic, lipophilic, or amphiphilic properties [79].

Ansari et al. aimed to create and evaluate a gel formulation based on ethosomes loaded with karanjin for improved topical administration and the successful treatment of skin acne [49]. Karanjin-loaded ethosomes had a nanometric size, reasonable entrapment efficiency and increased penetration by 1.9 times and skin deposition by 2.4 times compared to the hydro-ethanolic solution of drug. To ensure proper application on the skin’s surface, the created ethosomes were included in the carbopol gel. Confocal scanning laser microscopy (CLSM) showed a better penetration of karanjin-loaded ethosomal gel through rat skin. The karanjin-loaded ethosomal gel has significant antibacterial activity with a maximum zone of inhibition. Moreover, karanjin-loaded ethosomal gel exhibited good anti-inflammatory efficiency using a carrageenan-induced rat paw edema technique. Generally, ethosomal gels may be a good carrier method for topical karanjin administration in acne therapy.

Ferrara et al. examined ethosomes for curcumin (CUR) and piperine (PIP) administration to protect skin from diesel particulate matter, which is one of the most harmful environmental stressors [50]. Environmental contaminants can trigger oxidative and inflammatory responses in the skin, causing structural damage and premature aging. Curcumin is anti-cancer, anti-bacterial, anti-inflammatory, and anti-oxidant. Due to poor absorption and quick metabolism and excretion, it has limited bioavailability. Encapsulating curcumin in nanotechnology and adding biopotentiators like piperine improves its pharmacokinetics, stability, and action. Ethosomes were synthesized using several methods (cold method or by a microfluidic approach). Results showed ethosomes increase curcumin’s skin penetration. Studies showed that loading curcumin and piperine independently in ethosomes increased curcumin’s antioxidant potential, suggesting that they might protect skin damage from external stimuli. This study suggested that curcumin and piperine may regulate multiple inflammatory and oxidative stress pathways, protecting the skin from harmful environmental stressors.

Paolino et al. aimed to evaluate the ethosomal dispersion of water, phospholipids, and ethanol at various concentrations for the dermal administration of ammonium glycyrrhizinate, which is a drug used to treat inflammatory skin diseases [51]. Ethosomes were physicochemically characterized using photon correlation spectroscopy and freeze fracture electron microscopy. Franz’s cells were used to investigate the percutaneous penetration of ammonium glycyrrhizinate/ethosomes through human stratum corneum and epidermal membranes in vitro compared to drug solutions in water or in water–ethanol mixtures. In addition, the anti-inflammatory efficacy was assessed in vivo on human volunteers with cutaneous erythema. The developed ethosomes showed aresonable vesicle size and good entrapment efficiency. Human volunteers reported good skin tolerability with the ethosomal formulation even after 48 h. Ethosomes increased methylnicotinate and ammonium glycyrrhizinate in vitro percutaneous permeability. Ethosomes enhanced ammonium glycyrrhizinate’s anti-inflammatory efficacy compared to drug ethanolic or aqueous solutions. Results from in vivo investigations indicated that ethosomes could assure skin drug accumulation and sustained ammonium glycyrrhizinate release.

Abdallah et al. formulated ethosomal gel loaded with brucine to improve and assess its anti-inflammatory capabilities [52]. Brucine (BRU)-loaded ethosomal formulations were made by the thin film hydration method and then optimized by the central composite design approach with three response variables (vesicular size, encapsulation efficiency, and skin penetration) and three independent factors (lecithin concentration, cholesterol concentration, and ethanol percentage). The optimized formulation was then added to hydroxypropyl methyl cellulose (HPMC) gel to create brucine ethosomal gel. The resulting brucine-loaded ethosomal gel was assessed for physical characteristics, in vitro release, skin irritation, and ex vivo penetration. Ultimately, the anti-inflammatory effect was tested using the rat hind paw edema test caused by carrageenan. The produced brucine ethosomal gel showed good physical properties that were in accordance with the traditional brucine gel. For six hours, the in vitro release of brucine (BRU) from ethosomal gel was successfully prolonged. The ethosomes exhibited a considerably greater penetration of brucine compared to all other formulations, as evidenced by their steady-state transdermal flux value and enhancement ratio. The study concluded that ethosomal gel has the potential to be a promising carrier for enhancing the anti-inflammatory effect of brucine as evidenced by a significant decrease in rat hind paw inflammation after 24 h.

Alam et al. developed the ethosomal gel loaded with *Punica granatum* extract to enhance the penetration of its primary bioactive component, β-sitosterol transdermally [53]. In vitro and in vivo investigations were conducted on the created formulations. Using albino rats, the gel’s anti-inflammatory properties were evaluated. The formulation was optimized using the central composite design (CCD) with two dependent responses, particle size (nm) and entrapment effectiveness (%) and two independent variables like ethanol (mL) and lecithin (%). The generated ethosomes was characterized, and the mean zeta potential (−45.4 mV) and particle size (516.4 nm) were found, which demonstrated the stability of the developed nanoformulation (ethosomes) and their ability to cross the skin barrier. Assessments of the gel formulation were carried out. The gel’s formulation produced positive anti-inflammatory effects when applied to formalin-induced edema in albino rats. *Punica granatum* extract and gel were compared for their anti-inflammatory efficacy, and the results indicated that the gel’s action was greater for topical use.

### 2.3. Transethosomes

**Figure 5:**
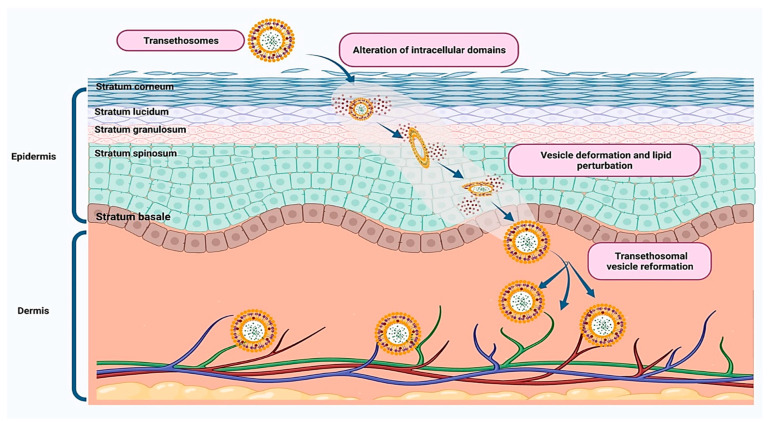
The transdermal pathway of transethosomes through the skin [84].

As a consequence of the considerable study that was conducted and published on transfersomes and ethosomes, a new generation of nanovesicular carriers emerged, which includes transethosomes, and glycerosomes. In recent years, these unique ultra-deformable vesicular systems have garnered interest [75,80]. Transethosomes, phospholipid nanovesicular carriers, combine ethosomes and transfersomes characteristics with ≥20% ethanol concentration and edge activators. Transethosomes are novel nanovesicular structures that resemble ethosomes (phospholipids, ethanol, and water) but include an edge activator or surfactant (penetration enhancer). They benefit from transfersomes and ethosomes [81,82]. Transethosomes promote medication penetration and bioavailability across skin barriers, permitting tailored administration to particular skin tissues (Figure 5). During skin permeation, the nanovesicular structure of transethosmes is maintained through deformation and reformation. The deformation of the lipid bilayer caused by an edge activator plays an essential role in the transdermal application of transethosomes [83].

Drug delivery to the target site of action reduces the risk of drug toxicity by lowering drug concentration in other body areas. Ethanol and surfactant increased penetration and softness, promoting passing across biological membranes, as determined by deformability and permeation studies [49]. They maintain prolonged medication release, lowering the administration frequency and unwanted side effects [84]. Transethosomes are ultra-flexible, with an elevated flux rate, and have great skin permeability compared to other vesicles. They are biocompatible, biodegradable, and highly stable with great patient compliance [85]. Transethosomes can capture both hydrophilic and lipophilic drugs/agents in the aqueous or lipid bilayer. Additionally, this vesicular structure is easily scalable and a great industry choice. However, because transethosomes have high alcohol content, they may induce skin dermatitis like ethosomes [81].

Jardan et al. created sinapic acid–transethosomes utilizing a thin-film hydration technique [54]. Sinapic acid is a bioactive phenolic acid with anti-inflammatory, antioxidant, anticancer, and antibacterial effects. The formulations were tested for several characterizations as surface morphology, in vitro penetration over the Strat M^®^, and antioxidant activity. The sinapic acid–transethosomes had smooth, spherical vesicles with appropriate entrapment efficiency. Sinapic acid–transethosomes also have increased antioxidant activity. The sinapic acid–transethosomes penetrated the Strat M^®^ membrane better than the control. The nano-sized vesicles and flexibility of sinapic acid–transethosomes could increase the drug penetration resulting from the incorporation of ethanol in transethosome nanovesicles. Both surfactant and ethanol in sinapic acid–transethosomes help the drug diffuse into the membrane. Thus, sinapic acid–transethosome-based techniques may enhance drug skin penetrability.

Moolakkadath et al. optimized the formulation of transethosomes for the topical administration of fisetin [55] using a Box–Behnken design, testing for vesicle size, entrapment, and skin penetration in vitro. The optimized formulation was used for vesicle–skin interaction, confocal laser scanning microscopy, and dermatokinetics. The optimized formulation had nano-sized vesicles, high entrapment efficiency, and adequate flux for fisetin dermal administration. Confocal investigation showed Rhodamine B-loaded fisetin transethosomes penetrated rat skin better than the control solution. Another dermatokinetic investigation found that fisetin transethosomes gel penetrated better than standard gel. The thermoanalytical results showed that the transethosome vesicles formulation fluidized the rat’s epidermal membrane, enabling enhanced fisetin transethosome penetration. Generally, transethosome vesicles were proven to be a promising medication carrier for fisetin cutaneous administration in this investigation.

Hassan et al., constructed transethosomes as nanovehicles for improved transdermal ginger extract delivery [56]. Ginger extract (GE) is known for its biological effects. Its low skin permeability hinders transdermal use. Cold injection with various edge activators produced GE–loaded transethosomes. Nanovesicles were assessed for their particle size, zeta potential, encapsulation effectiveness, and in vitro drug release. The optimized formulation was incorporated into different gelling agents, chitosan (2% *w*:*v*), hydroxypropyl methylcellulose (HPMC; 3% *w*:*v*), and sodium alginate (4% *w*:*v*) to produce a transethosomes hydrogel system and then tested for ex vivo permeability and in vivo anti-inflammatory efficacy utilizing a carrageenan-induced rat-paw edema model. The results demonstrated that transfersomal gel containing HPMC showed better transdermal flux, skin permeability, and skin deposition compared to plain gel and a free drug. In vivo and histological tests showed that ginger extract transethosomal hydrogel inhibited edema swelling better than free ginger extract hydrogel and standard anti-inflammatory ketoprofen gel. Generally, ginger extract transethosomes could be an acceptable skin permeation and anti-inflammation carrier.

Adin et al. generated baicalin-loaded transethosomes (BCL-TE) to increase transdermal Baicalin solubility, bioavailability, and skin permeability [57]. Thin-film hydration was used to create baicalin-loaded transethosomes and optimized utilizing a Box–Behnken design. The optimized baicalin-loaded transethosomes were tested for PDI, vesicle size, transmission electron microscope (TEM), entrapment effectiveness, zeta potential, and in vitro BCL release. Pharmacokinetic, skin permeation and confocal scanning laser microscopy (CLSM) studies were conducted for further investigation. The baicalin-loaded transethosomes had spherical, smooth vesicles that had a 141.2 nm size, 76.39% entrapment efficiency, 0.1135 PDI, and 65.32% in vitro release. The confocal scanning laser microscopy (CLSM) investigation found that the formulation permeates baicalin across skin layers better than the plain BCL gel. The study found that the transdermal delivery of baicalin-loaded transethosomal gel resulted in a higher C_max_ and AUC _0–24_ compared to oral administration. The baicalin-loaded transethosomes gel has better anti-arthritic potential than standard anti-inflammatory diclofenac gel in vivo, as shown by radiographic and histological tests. Additionally, Wistar albino rats’ skin irritation studies show that the baicalin-loaded transethosomes formulation is safe for skin use. Consequently, the study confirmed that the transethosome vesicle formulation is a valuable carrier for baicalin transdermal administration for rheumatoid arthritis.

Abdulbaqi et al. studied the transethosomal gels for transdermal colchicine administration to alleviate the drawbacks of colchicine such as its low bioavailability, significant gastrointestinal adverse effects, and poor therapeutic index [58]. Colchicine-loaded transethosomes (TEs) were prepared by cold methods and statistically optimized utilizing factorial design. The optimized formula was incorporated into a Carbopol 940^®^ gel base. The analyzed properties of colchicine-loaded transethosomal gels included their vesicular size, polydispersity, zeta potential, drug content, pH, viscosity, yield, rheological behavior, and ex vivo skin penetration via Sprague–Dawley rats’ back skin. Colchicine-loaded transfersomes exhibited an aspherical form, nanometric size range, and excellent entrapment efficiency. Colchicine-loaded transethosomal gels improved medication skin penetration compared to non-ethosomal gels. These findings revealed that transethosomal gels might administer colchicine transdermally, offering an alternate medication delivery route.

### 2.4. Glycerosomes

Glycerosomes are innovative vesicular drug delivery vehicles that may be used topically or systemically [86]. These noninvasive carriers have the ability to reach deeper skin layers than a free medication could. Glycerosomes are composed of phospholipids, water, and a significant amount of glycerol (20–40%), which make glycerosomes safe and suitable components for topical formulations [60,87,88]. Higher drug penetrations are made by high glycerol concentrations, which also improve the vesicle stability and flexibility [88]. These flexible vesicles can include a variety of substances, such as cholesterol, which improves the lipid bilayer’s stability. Moreover, to reduce vesicle aggregation and alter the electrical charge of the vesicular surfaces, basic or acidic lipid molecules can be added. These vesicles have better spreadability and penetrability because of their high glycerol content, and they are biocompatible. Therefore, it is essential to investigate alternative topical medication delivery methods, including ophthalmic, vaginal, nasal, and rectal [89]. Glycerosomes are a special type of lipid-based vesicle, similar to liposomes, and these are used as carriers for delivering drugs or bioactive compounds. They are composed of glycerolipids, which are natural components of cell membranes, making them biocompatible and suitable for drug delivery applications [90]. Loading natural drugs or bioactive compounds into glycerosomes offers several advantages. For instance, natural drugs often have fewer side effects compared to synthetic ones, and they may also possess anti-inflammatory properties. By encapsulating these compounds within glycerosomes, their stability can be enhanced, and their targeted delivery to inflamed tissues can be facilitated [91]. In the context of inflammation, glycerosomes loaded with natural drugs can be designed to target specific inflammatory pathways or cells, reducing inflammation at the site of action while minimizing systemic side effects. This targeted delivery approach is particularly advantageous for chronic inflammatory conditions like arthritis, where localized treatment is preferred [92]. Furthermore, the use of natural drugs may offer additional benefits such as antioxidant effects and the modulation of immune responses, which can contribute to the overall efficacy of the treatment [93]. Overall, utilizing glycerosomes loaded with natural drugs for inflammation represents a promising approach in drug delivery, offering the potential for enhanced therapeutic outcomes with reduced adverse effects. However, like any therapeutic intervention, further research is needed to optimize formulations and evaluate their efficacy and safety in clinical settings. Moreover, they can encapsulate both hydrophilic and lipophilic medicines and shield them from deterioration. They can be obtained by any of the different techniques commonly used for the preparation of conventional liposomes. The structure, benefits, makeup, preparation techniques, characterization, and uses of glycerosomes are outlined in this review article.

Zhang et al. optimized paeoniflorin’s (PF) transdermal drug delivery and synovial drug absorption by the development of the glycerosomes carrier including essential oils for topical administration [59]. Glycerosomes were generated applying a reverse-phase evaporation technique using different concentrations of phospholipid, cholesterol and glycerin and 2% (*v*/*v*) speranskia tuberculata essential oil (STO) as a transdermal enhancer to enhance the permeation of paeoniflorin via the skin. The results revealed that the in vitro transdermal flux of paeoniflorin loaded in STO–glycerosomes was greater than the corresponding liposomes and drug tincture. According to in vivo investigation, using STO–glycerosomes led to obtaining a higher amount of paeoniflorin accumulation in the synovium than using plain glycerosomes. In vivo imaging experiments supported this discovery by demonstrating that 5 h after injection, the fluorescence intensity of Cy5.5-loaded STO-glycerosomes in mice’s knee joints was 1.8 times greater than that of ordinary glycerosomes. Significant skin permeability and enhanced drug absorption in the synovium were demonstrated by the STO-mediated glycerosomes, suggesting that STO-glycerosomes might be a viable paeoniflorin transdermal delivery vehicle for the treatment of rheumatoid arthritis brought on by synovium lesions.

Alam et al. developed and created carbopol gel-encased glycerosomes for the treatment of sunburn [60]. Glycerosomes loaded with rutin were prepared using a thin-film hydration method followed by sonication. The formulation comprised phospholipids, cholesterol, and glycerol, and it was optimized using the Design of Expert (DoE) technique to encapsulate rutin efficiently. The polydispersity index (PDI), nanovesicles size, zeta potential, surface morphology and drug encapsulation efficiency were characterized using dynamic light scattering, ultracentrifugation and spectroscopic techniques. Glycerosome-encapsulated rutin exhibited a narrow size distribution (approximately 123.7 nm) and a negative surface charge indicated by the negative zeta potential value, facilitating stability and cellular uptake. The encapsulation efficiency of rutin was found to be high: about 82.81%. In vitro studies demonstrated a sustained release of rutin from glycerosomes with enhanced stability in physiological conditions. Additionally, the optimized glycerosomal formulation was incorporated into carbopol 934P gel. A dermatokinetics research revealed that glycerosomal gel exhibited superior penetration compared to conventional gel. In addition, the formulation was evaluated for its potential in treating inflammation in sunburn and showed efficacy in treating sunburn with a desired sun protection factor (SPF) value and excellent antioxidant capabilities. The formulation has been deemed appropriate for safe use by topical distribution, as verified by a skin irritation study. The results have shown that the developed glycerosomal gel had the capability to exhibit stability for extended durations when kept under standard conditions.

### 2.5. Bilosomes

Bilosomes are specialized lipid-based vesicles designed for enhanced drug delivery, similar to liposomes and glycerosomes. They are composed of bile salts and phospholipids, which self-assemble into bilayer structures capable of encapsulating drugs or bioactive compounds. They possess a distinctive ability to permeate biological membranes, including those found in the skin and intestine [94,95]. The presence of bile salts hinders the breakdown of nanocarriers in the gastrointestinal system, resulting in improved penetration, which is advantageous for oral administration [96]. Furthermore, the transdermal administration is enhanced because of the exceptional flexibility of bilosomes, resulting in improved penetration into the stratum corneum and deeper layers of the skin. In addition, the incorporation of certain bile salts, such as sodium deoxycholate, enhances the colloidal stability of the system as compared to conventional liposomes. Additionally, it possesses a diameter at the nanoscale and exerts a fluidizing impact, hence augmenting transdermal administration [97]. Bilosomes have gained attention in recent years as promising carriers for delivering drugs or natural products, including those with anti-inflammatory properties, to target sites within the body. Bilosomes loaded with natural anti-inflammatory drugs offer a promising therapeutic strategy for managing inflammation, providing targeted delivery, improved bioavailability, and reduced systemic side effects [98].

Elkomy et al. formulated chitosan-coated bilosomes nanogel to enhance the transdermal delivery of berberine [61]. This approach was intended to improve the treatment of inflammation associated with rheumatoid arthritis. The formulation of chitosan-coated bilosomes loaded with berberine was carried out using the thin-film hydration method. The formulation was optimized using the Box–Behnken design, considering different related variables such as lipid concentration, sodium deoxycholate concentration, and chitosan concentration. The aim was to investigate the impact of these variables on the particle size, entrapment efficiency, and surface charge of the bilosomes. The optimized bilosomes exhibited a mean diameter of 202.3 nm, entrapment efficiency of 83.8%, and surface charge of 30.8 mV. The optimized formulation had a delayed-release pattern in vitro and showed an enhanced capability to penetrate the skin, which was proven by ex vivo permeation studies. The optimized bilosomal formulation was then combined with a Carbopol 974 NF polymer in order to create a nanogel. The histological analysis demonstrated that the prepared biolosomes did not cause any skin irritation. In addition, the efficacy of the tailored chitosan-coated bilosomes loaded with berberine in reducing inflammation was assessed in rats with carrageenan-induced paw edema. The findings of this study show that the group treated with topical chitosan-coated berberine bilosomes gel showed a significant decrease in rat paw edema swelling percentage, reaching 24.4% after 12 h. This reduction was considerably more than that observed in the other groups treated with Voltaren emulgel (reference standard) and topical berberine gel. Chitosan-coated bilosomes containing berberine have shown promise as a therapeutic method for controlling inflammation in rheumatoid arthritis.

### 2.6. Phytosomes

**Figure 6:**
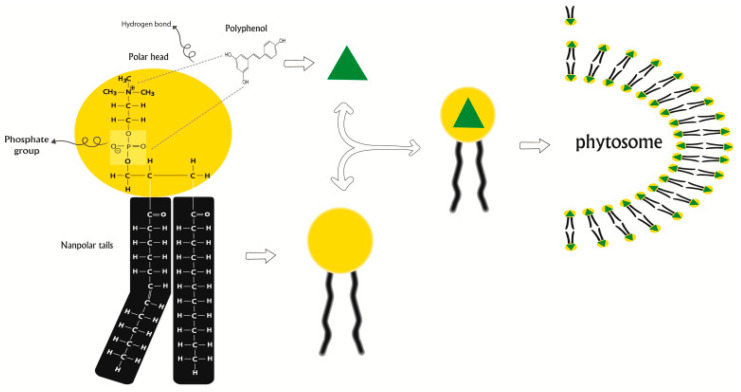
Proposed concept for the production of phytosomes. The creation of hydrogen bonds between phytochemicals and the polar head of phospholipids is illustrated through schematic and structural images. Dashed lines symbolize the hydrogen bonds [105].

The name “Phyto” pertains to plants, whereas “some” pertains to structures resembling cells [99]. Bombardelli et al. were the first to establish the existence of a chemical connection between phospholipids and flavonoid vegetal derivative molecules [100]. Phytosomes, also known as herbosomes, are a type of drug delivery system that improves the absorption and bioavailability of medications with limited solubility by using vesicles [101]. Phytosomes are intricate combinations of phospholipids and natural active phytochemicals (Figure 6). They are formed by reacting phosphatidylcholine (or similar hydrophilic polar head groups) with plant extracts in a solvent (aprotic solvent) [102]. These formulations demonstrate enhanced pharmacological and pharmacokinetic characteristics in comparison to conventional preparations. The hydrophilic phytoconstituent–choline complexes are fully enveloped by the lipid-soluble phosphatidyl part. Phytosomes provide notable advantages, including a large capacity for medication encapsulation, improved stability (achieved by chemical bonding between the polar head of the phytoconstituent and amphiphile molecule) [103], and enhanced bioavailability [104]. Furthermore, a greater rate of absorption results in a reduced amount of active components needed to provide a biological impact, particularly for polar phytoconstituents [105,106].

Several studies have proven that phytosomes have superior anti-inflammatory properties compared to the purified extract of herbal materials. An investigation was carried out to examine the absorption of rutin phytosomes into the skin. The results revealed that rutin phytosomes have superior ability to enter the extremely impermeable stratum corneum compared to free rutin. The increased amount of rutin will be retained for a longer period of time as it slowly passes through the viable dermis, resulting in a sustained anti-inflammatory action [62]. The anti-inflammatory efficacy was evaluated using carrageenan-induced inflammation on rats; the test group treated with rutin phytosomes showed a substantial reduction in inflammation compared to the group treated with the standard anti-inflammatory diclofenac gel. Rutin phytosomes, being lipophilic, are deposited in the epidermal–dermal area, resulting in a delayed release of the drug and providing a prolonged anti-inflammatory action [107]. Ho et al. showed that the inflammatory symptoms caused by pthalic anhydride (PA) induce atopic dermatitis (AD) in a mouse model, such as redness, swelling, and skin damage on the ears and back, were significantly reduced following treatment with *Centella asiatica* phytosomes. This effect was observed when comparing the treatment group to both the control group and the group that received just PA treatment [63]. Another study found that the lawsone phytosome gel treatment had notable anti-inflammatory effects compared to the plant lawson gel after 4 h in rats with carrageenan-induced paw swelling [64].

Ramachandran et al. focused on the development and assessment of a herbal gel containing a methanolic extract of *Crotalaria biflora* topically to enhance its anti-inflammatory properties [65]. The preparation of extracts from the whole plant was carried out using methanol as the solvent through a process called maceration. The *Crotalaria biflora* phytosome was synthesized utilizing the rotary evaporation method, combining a plant extract with phosphatidylcholine. Several investigations were carried out on the phytosomal formulation, including organoleptic assessment, entrapment efficiency, and Fourier-transform infrared spectroscopy (FTIR) examinations. The phytosome was developed as a gel and analyzed for many physicochemical properties including uniformity, pH level, viscosity, spreadability, and extrudability. Additionally, a diffusion study and in vitro assessment of anti-inflammatory activities were conducted. Based on the research findings, it was determined that the methanolic extract of *Crotalaria biflora* may be effectively used to create a herbal topical phytosomal gel with enhanced anti-inflammatory efficiency. This gel has demonstrated notable effectiveness in reducing inflammation when applied topically.

## 3. Conclusions

Exploring new drug delivery systems is essential to study the various therapeutic properties of naturally occurring medications. These approaches are limited by lower water solubility and bioavailability. Researchers have devised novel techniques for drug delivery, either by encapsulating the medication within a carrier or by modifying the molecule’s structure by the addition of stabilizing groups. The most important thing to keep in mind when creating any formulation is that it must be produced in order to pass through biological membranes. The primary factors considered are the drug’s lipid solubilities and molecular size. Recent studies have forecasted the potential use of nanovesicular gel loaded with natural drugs in treating several diseases, including inflammation through skin. Current research is focused on overcoming existing obstacles by employing nanovesicular techniques for natural products delivery via skin. Nanocarriers achieved a diminished drug concentration in the bloodstream, leading to less toxicity, which is beneficial for patients who need daily treatment. Pharmaceutical nanotechnology is a rapidly developing scientific discipline that focuses on enhancing the stability, solubility, absorption, and bioavailability of pharmaceuticals that have low water solubility and limited bioavailability. In addition, nanotechnology-based solutions improve the precise and prolonged distribution of the trapped substance, resulting in effective therapeutic strength while minimizing adverse effects. The prevalence of inflammatory disorders and their urgent care underscores the necessity for developing advanced systems that will improve therapeutic efficacy while avoiding the drawbacks of traditional methods. Advanced nanovesicular carriers are innovative drug delivery technologies that enhance the transportation of natural drugs via the skin. By specifically directing the medicinal agents to the intended site of action and reducing their adverse effects, their therapeutic effectiveness in treating inflammatory illnesses can be enhanced. Each nanovesicular system contains a unique component that promotes the capacity of drugs to penetrate the skin and increases their bioavailability. This study provides a summary of previous research as well as the development of novel nanovesicular gel formulations as delivery system through the skin, with a particular emphasis on natural products used for the treatment of inflammation. Formulation and development may greatly enhance the effectiveness, stability, and shelf life natural products.

At present, attention should be on advancing the commercial development of bioactive natural products. In order to achieve an economical formulation, it is necessary to minimize the cost of the delivery system. It is necessary to explore the current market and research progress of the innovative delivery development system for natural products in order to explore its potential for various therapeutic agents. In the future, it is possible to isolate and examine these natural products for their pharmacological and therapeutic effects using in vivo models. Nevertheless, it is possible to transform traditional natural products into vesicular carrier drug delivery systems, resulting in enhanced physicochemical properties, pharmacokinetics, and pharmacodynamics. Thus, it is anticipated that the unique structural characteristics and qualities of the new nanovesicular carriers would enhance the effectiveness of natural compounds in the future, resulting in significant benefits for humanity.

## Figures and Tables

**Table 1:** Advanced nanovesicular drug delivery systems for effective delivery of plant-based anti-inflammatory compounds.

Nanovesicular System	Plant-Based Compounds	Composition	Method of Preparation	Special Characteristics	References
Transfersomes	Resveratrol	Lecithin and edge activators (Tween 20, Tween 80	High-pressure homogenization technique	High flexibility and stability, enhanced skin permeability and improved solubility, bioavailability, and safety of drug	[44]
	Curcumin	Lecithin and edge activators (Tween 80, Span 80	Modified hand shaking, lipid film hydration technique	Improved skin permeability of drug	[45]
	Sesamol	Phosphatidylcholine, Tween 80 and Span 80 edge activators	Thin-film hydration	Better skin penetration and deposition were observed in sesamol-loaded transfersomal gel	[46]
	Quercetin	Phosphatidylcholine and tween 80	Thin lipid film hydration technique	Quercetin-loaded transfersomes were found to be a good alternative to oral administration of quercetin to treat osteoporosis	[47]
	*Berberis aristata* extract	Soyaphosphatidylcholine (SPC), edge activator (EA Tween 80/Span 80/sodium deoxycholate)	Modified lipid film hydration technique using rotary evaporator	Better therapeutic efficiency	[48]
Ethosomes	Karanjin	Phospholipids 90 G, ethanol and phosphate buffer	Film hydration method	Greater flexibility, enhanced skin permeation and effective anti-inflammatory activity	[49]
	Curcumin and piperine	Soybean lecithin, ethanol, water	Bulk cold method or by a microfluidic approach	Prolonged transdermal release of the drugs within the skin	[50]
	Ammonium glycyrrhizinate	Phospholipids 90 G, ethanol and water	Film hydration method	Higher percutaneous permeation and enhanced anti-inflammatory activity	[51]
	Brucine	Lecithin, cholesterol, ethanol and phosphate buffer pH 7.4	Thin film hydration method	Greater skin permeability and higher anti-inflammatory activity	[52]
	*Punica granatum* extract	Lecithin, cholesterol, propylene glycol, ethanol, and water	Hot method followed by sonication or extrusion techniques	Greater potential of *P. granatum* ethosomal gel for enhancing its anti-inflammatory activity	[53]
Transethosomes	Sinapic acid	Phospholipon 90 G, SDC, ethanol, phosphate-buffered saline	Thin film hydration method	Enhanced penetrability across the membrane, and improved vesicles flexibility	[54]
	Fisetin	Lipoid S 100, sodium cholate and ethanol	Thin lipid film hydration technique	Deeper skin penetration and deposition	[55]
	Ginger extract	Phospholipon 90 G, cholesterol, edge activator (Span 80, Tween 80, and sodium deoxycholate) and ethanol	Cold injection technique		[56]
	Baicalin	Phospholipon 90 G, Sodium cholate, ethanol	Thin film hydration method	High elasticity and skin deposition	[57]
	Colchicine	Phospholipon 90 G and surfactant (Tween 20^®^, sodium taurocholate, or Labrafil^®^) in ethanol	Cold method	Transfersomes is proven to be an alternative route to the oral route to overcome bioavailability problems and other side effects	[58]
Glycerosomes	Paeoniflorin	Lipoid S 80, cholesterol, water and glycerol	Reverse-phase evaporation method	Superior transdermal flux, safe and applicable vehicle for the treatment of rheumatoid arthritis	[59]
	Rutin	Phospholipid 90 G, cholesterol, water and glycerol	Thin film hydration method	Suitable alternation for administration of drug topically to maximize the therapeutic efficacy of the drugs	[60]
Bilosomes	Berberine chloride	Cholesterol, soybean lecithin, sodium deoxycholate	Thin-film hydration technique	Chitosan-coated bilosomes, which contain berberine, have shown promise as a therapeutic method for managing inflammation in rheumatoid arthritis (RA).	[61]
Phytosomes	Rutin	Phosphatidylcholine	Refluxing followed by solvent evaporation	Rutin phytosomes improve skin absorption to treat inflammatory disorders and supply the medicine longer without oral administration complications	[62]
	Centella asiatica	Phospholipid such as phosphatidylcholine, phophatidylethanolamine or phosphatidylserine, (dioxane, acetone, methylene chloride, or ethyl acetate)	Solvent evaporation, precipitation and anhydrous co-solvent lyophilization	Phytosome effectively reduces both skin inflammation and PA treatment-induced allergic responses	[63]
	*Lawsonia inermis* L. (Lawsone)	Lawsone and soya lecithin	Anti-solvent precipitation technique	The anti-inflammatory activity of lawsone phytosomal gel showed significant anti-inflammatory activity as compared to lawsone gel	[64]
	Methanolic extract of *Crotalaria biflora*	Plant extract with phosphatidylcholine	Rotary evaporation method	Development of herbal topical phytosomal gel with enhanced anti-inflammatory efficiency	[65]

## Data Availability

Not applicable.
